# Design of a Multi-Point Kinematic Coupling for a High Precision Telescopic Simultaneous Measurement System

**DOI:** 10.3390/s21196365

**Published:** 2021-09-23

**Authors:** Raquel Acero, Juan José Aguilar, Francisco Javier Brosed, Jorge Santolaria, Sergio Aguado, Marcos Pueo

**Affiliations:** 1 Department of Design and Manufacturing Engineering, University of Zaragoza, María de Luna 3, 50018 Zaragoza, Spain; jaguilar@unizar.es (J.J.A.); fjbrosed@unizar.es (F.J.B.); jsmazo@unizar.es (J.S.); saguadoj@unizar.es (S.A.); 2 Instituto de Investigación en Ingeniería de Aragón (I3A), 50018 Zaragoza, Spain; 3 Centro Universitario de la Defensa, Academia General Militar, Ctra. Huesca s/n, 50090 Zaragoza, Spain; mpueo@unizar.es

**Keywords:** kinematic coupling, telescopic system, interferometry, machine tool, multilateration

## Abstract

This paper covers the design of a new multi-point kinematic coupling specially developed for a high precision multi-telescopic arm measurement system for the volumetric verification of machine tools with linear and/or rotary axes. The multipoint kinematic coupling allows the simultaneous operation of the three telescopic arms that are registered at the same time to a sphere fixed on the machine tool spindle nose. Every coupling provides an accurate multi-point contact to the sphere, avoiding collisions and interferences with the other two multi-point kinematic couplings, and generating repulsion forces among them to ensure the coupling’s fingers interlacing along the machine tool x/y/z travels in the verification process. Simulation presents minimal deformation of the kinematic coupling under load, assuring the precision of the sphere-to-sphere distance measurement. Experimental results are provided to show that the multi-point kinematic coupling developed has repeatability values below ±1.2 µm in the application.

## 1. Introduction

The design of interfaces and connections between parts can be generally achieved by kinematic and elastically averaged designs [1,2]. In an exact-constraint design, the freedom of motion is restricted, applying the minimum number of constraints required, meanwhile elastic averaging designs provide multiple overconstrained contact points [3]. Kinematic couplings are extensively used to locate parts with a high accuracy such as fixtures, testing equipment or positioning systems [4]. Considered as exact constraint design couplings, they use six known contact points to locate one component with respect to another, being the relationship between the surfaces described by closed loop mathematics [5]. They are an economical solution in design and manufacturing that allows obtaining a high repeatability [6,7]. Nevertheless, the surface finish and contamination of the contact regions could restrict the repeatability of kinematic couplings because of tangential forces entrapped by friction, while the load capacity and stiffness are limited by the Hertzian contact stress that appears between the components in the point or line of contact, therefore, all these factors being important to consider in the coupling design.

In addition, symmetry plays an important role in kinematic coupling designs. One could identify non-symmetric kinematic coupling designs such as the Kelvin coupling, where its instant center of rotation is always located at the center of the tetrahedron’s contact points. On the contrary, there are symmetric designs such as the common three-V-groove couplings or the Maxwell coupling with three-V grooves on one part oriented towards the center of the coupling, and three mating curved surfaces on the other part [8].

There are multiple types of kinematic couplings. Some of the most frequently used are spatial kinematic couplings such as three-groove kinematic couplings, quasi-kinematic couplings, compliant kinematic couplings, including flexural, split groove, planar couplings or three-tooth couplings forming three theoretical lines of contacts [5]. However, other approaches include servo-controlled kinematic couplings that use servo-controlled actuators to control the position of the coupling, or kinematic coupling designs that cope not only with positioning, but also with functional requirements such as fluid and electrical connections.

As explained in [9], the performance of a kinematic coupling depends on its geometry, materials, kinematic and thermodynamic properties. The variations in the inputs such as the force (momentum transfer to or between the coupled components), displacement (relative motion between the coupled components) or in the system characteristics (geometry or coupling properties) could generate variations in the output versus the expected one, which are accounted for as the repeatability of the kinematic coupling. In addition, the tolerances of the components of the coupling and their assembling directly affect the accuracy of kinematic couplings [10], being recommendable to control the deformation and the friction in the contact interface to improve the coupling’s repeatability.

Normal applications for kinematic couplings are such with light loads and static conditions, but corrosion-resistant materials used in contact areas could provide the kinematic coupling robustness for a high stiffness, high-cycle and high-load applications [11,12].

In this work, the design of a multi-point kinematic coupling to be assembled in a telescopic measurement system developed by the authors for machine tool (MT) volumetric verification is presented. The target application is a light load and quasi-static measurement, being the use of kinematic couplings suitable in this case.

Machine tool verification by an indirect error measurement such as volumetric verification [13,14,15,16,17,18,19] allows a mathematical, non-physical correction of the joint influence of geometric and kinematic errors of the machine on its entire working volume, while reducing the verification time used by conventional methods [20]. It is based on the multi-axis movement of the machine to reach the measurement points both for machines with three linear [21,22] and five axes, linear and rotary [23,24]. In either case, the volumetric verification is supported by an intensive error identification process for a non-linear model, based on positioning error data distributed in the working volume and an objective function of the error.

The telescopic system where the multi-point kinematic coupling is assembled, is based on simultaneous laser multilateration and focuses its application on the verification of small and medium-sized machine tools with 3/5 axes. It offers the high precision of interferometric systems and allows the autonomous tracking of a fixed sphere, either attached to the machine tool spindle nose or positioned on the MT bed for the verification of rotary axes, through the simultaneous contact of three telescopic lines using a novel multi-point kinematic coupling, whose development is presented in this work. Data capture is carried out in a single cycle thanks to the simultaneous operation of the three telescopic arms, providing a flexibility and measurement process time similar to those of the laser tracker, but improving its precision to levels close to those of laser interferometry, avoiding the effect of temperature variations between cycles.

The contents of the work are structured in the following way in the paper: Section 2 briefly presents the concept of the high precision telescopic system, the multi-point kinematic coupling and its location and functionality in the equipment. Section 3 discusses the working principle, requirements, design parameters and design versions considered and evaluated in the development process of the kinematic coupling. Section 4 provides experimental results of the prototypes which show that the multi-point kinematic coupling can provide repeatability values adequate for the intended application. The main conclusions of the work are covered in Section 5.

## 2. The High Precision Telescopic System

**Figure 1:**
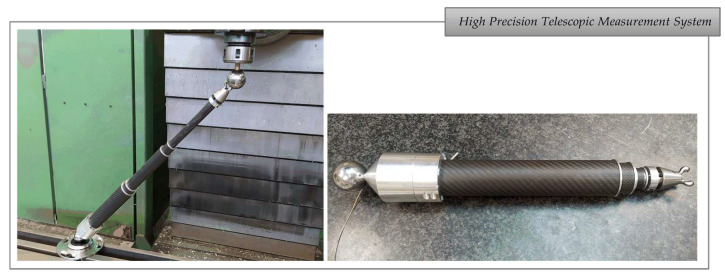
Extensible telescopic measurement system prototype.

The multi-point kinematic coupling was specially developed for a multi-telescopic arm measurement system based on multilateration to be used in the volumetric verification of small and medium-sized machine tools with linear and/or rotary axes [25]. The telescopic arms have a conventional kinematic support at one end, and a multi-point kinematic coupling at the other end, which connects to a common ferromagnetic sphere attached to the MT spindle nose (see Figure 1).

**Figure 2:**
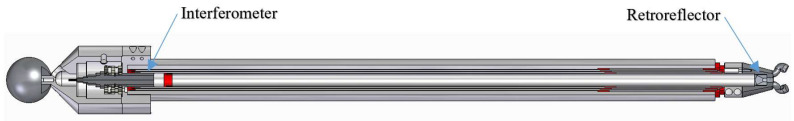
Interferometer and retroreflector positions.

The different lengths are materialized with a telescopic system composed of carbon fiber that is divided in several stages assuring the alignment of the interferometer and the retroreflector in its extension. The interferometer (see Figure 2) is located on one side of the telescopic system, close to the fixed sphere on the MT table, and the retroreflector is located on the other side of the system near to the sphere fixed to the MT spindle nose.

## 3. The Multi-Point Kinematic Coupling

### 3.1. Working Principle

The multi-point kinematic coupling, named trident in the work because of its trident-like shape, has three fingers that generate a multi-point contact with the fixed sphere attached to the MT spindle nose. This is achieved with three magnets located at the ends of the fingers. The registration to the sphere must not only be accurate, but should also consider the simultaneous registration of two additional tridents from the other two telescopic arms, which form the high precision measurement system. The trident needs to have enough force not to be detached from the fixed sphere along the MT travel in the different axis. It also needs to generate repulsion forces among the other trident’s fingers to avoid collisions or interferences that would hinder the correct working of the telescopic arms set. Minimal space is required between the fingers to ensure the correct movement of the system thanks to the interlacing fingers.

Each finger is located in the trident forming a relative angle between the axis of rotation of the telescopic system passing through the center of the fixed sphere and the line joining the magnet contact point and the center of the sphere.

### 3.2. Requirements of the System

In the design process of the multi-point kinematic coupling, several requirements and restrictions were considered because of their impact on the measurement precision and on the complete high precision telescopic system performance. These were mainly derived from the precision required in the measurement, the geometric restrictions and (x/y/z) travels of the MT where the telescopic system will be located in the volumetric verification, the maximum effective length to be measured considering the MT verification volume and from the design of the telescopic arm itself.

**Figure 3:**
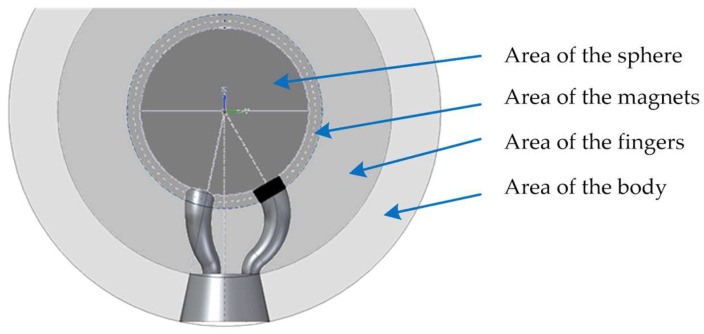
Areas of influence in the trident’s design.

These requirements could be classified into four main areas in the coupling design, which are the area of the fixed sphere attached to the MT spindle nose, the area of the magnets and the areas of the coupling’s fingers and body, respectively (see Figure 3).

In the area of the fingers, the aim is to assure their interlacing; meanwhile, in the area of the body, the design must allow the fingers to not collide up to a certain angle depending on the range of the MT to be verified. These requirements limit the design of the trident, especially in the area of the fingers, which must be short to minimize the distance between the fixed sphere attached to the MT spindle nose and the retroreflector. This allows maximizing the accuracy of the distance measurement. In addition, the fingers must insert into each other, preventing crashes by interlacing themselves. The geometry selected enables a minimum percentage of non-collision among the three tridents, which was consider as an input in the design process [25].

In relation to the parameters affecting the magnets area, it was ensured that the magnets repel each other and that they couple to the fixed sphere without loosening. The force of the trident’s magnets must be sufficient so that the trident does not detach from the fixed sphere due to the supported loads in the static measurement, which are the inner weight of the kinematic coupling and the weight of the telescopic arms, 855 grs in total. This fact comes directly in relation with the selection of the magnets’ parameters (diameter, height and force) considering the fixed sphere’s diameter definition. Inertia forces also have to be taken into account here, when moving from point to point at high feed rates (up to 20 m/min) in the MT.

The requirements on the sphere’s area are related to the diameter of the sphere and its roundness to give accuracy to the measuring system. The fixed sphere diameter varied between 1.5 inches and 2 inches in the different designs.

Finally, it was very important to minimize the deformation of the trident which could lead to a relative movement between the center of the sphere and the trident, as this would include an error in the laser measurement of the sphere-to-sphere distance.

A reduced-size kinematic coupling was designed that permits the housing of the magnets, accommodates the retroreflector and includes a fixing area to the carbon fiber tube. The final design of the kinematic coupling was mechanized in stainless steel grade 304 L in a single part, but different approaches were considered in the design phase, trying to minimize the number of components of the coupling as presented in the next section.

### 3.3. Kinematic Coupling Design Parameters

The design process of the trident started by considering the main requirements and restrictions of the complete high precision telescopic measurement system described in the section before. Based on these, different approaches for the design concept were evaluated.

**Figure 4:**
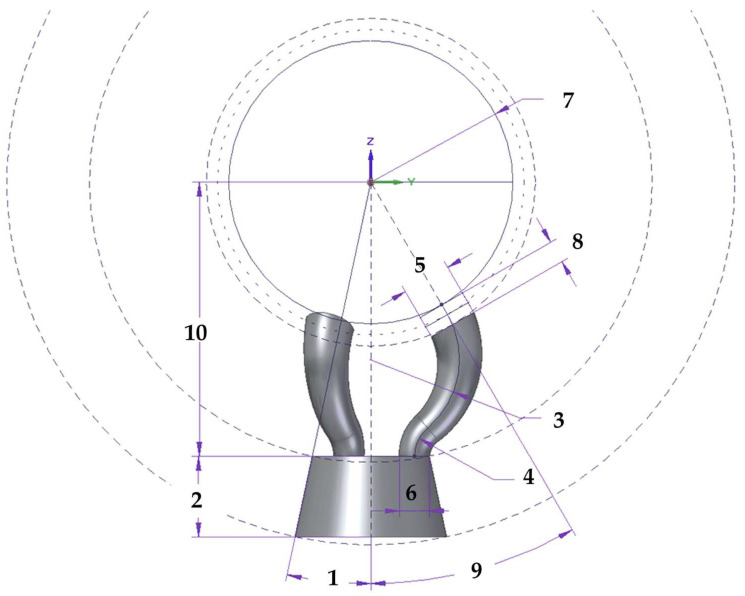
Multi-point kinematic coupling design parameters and areas.

The multi-point kinematic coupling’s design parameters were structured into two main groups as described in Table 1. Group one considers the parameters related to the main parts or areas of the trident geometry (fingers and body). The second group includes parameters related to other components affecting the multi-point kinematic coupling such as the fixed sphere diameter, the magnets’ diameter and height, the contact point angle and the distance between the fixed sphere center and trident’s body. Figure 4 shows the definition and location of the most relevant design parameters of the multi-point kinematic coupling.

#### 3.3.1. Tridents Magnets and Sphere Diameter Relation

The trident has three fingers that contact the sphere precisely at various points, forming a given relative angle from the center of the sphere (ID 9). To avoid possible collisions among the three tridents and allow their free movement, magnets are used with a location at the end of the trident’s fingers. The finger’s magnet parameters (diameter (ID 5), height (ID 8) and force), as well as the geometry of the trident’s fingers, have a direct relation to the diameter of the fixed sphere (ID 7). The magnets generate attraction forces that maintain the tridents attached to the sphere and repulsion ones that prevent collisions during the MT verification travels. According to the simulation performed in [25], a fixed sphere of 1.5 inches in diameter and a trident’s finger diameter of 6.5 mm were selected, which led to a cylindrical neodymium magnet of 6.5 mm in diameter and 3 mm in height with 2.2 kg of force.

It was empirically verified in measurement tests with a rapid MT feed x/y/z of about 20 m/min, where inertial forces are also important, that the system operates without the kinematic couplings being released from the fixed sphere attached to the MT spindle nose.

#### 3.3.2. Contact Point Angle

The contact point angle (ID9) is defined as the relative angle formed by the rotation axis of the telescopic system passing through the center of the sphere and the line joining the magnet contact point and the center of the fixed sphere attached to the MT spindle nose.

Three different tests were carried out in order to determine the forces supported by the magnets and the optimum contact point angle evaluating several trident’s designs.

The first test was a static test, and evaluated the force needed to detach a single metallic sphere of 1.5” in diameter from the trident, pulling directly from the sphere and applying an axial force in the direction of the telescopic arm.

The values obtained in the static test evaluating eight different trident’s geometries with different contact point angles, varying from 25 to 50 degrees, each five degrees, and adding a last case at 54.74 degrees that corresponded to the position of the contact points that formed an inverted 90 degree trihedron. The results showed that the highest forces would withstand the 25 degree contact point angle geometry, with a value of 1.04 kg as shown in the results in Table 2.

**Figure 5:**
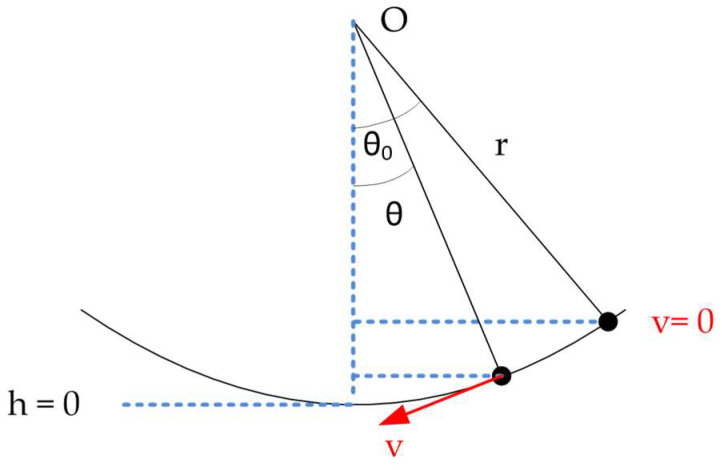
Dynamic test—pendulum test scheme.

The second and the third tests were dynamic tests, and used a simple pendulum method with a test bench. The target was to evaluate with different contact point angles if the magnets supported the force of the pendulum launch without loosening the trident from the fixed sphere. With the launch angle known, (θ_0_) as shown in Figure 5, the maximum velocity of the pendulum (v) was calculated by equating the maximum potential and kinetic energies. The maximum potential energy was produced at the initial position before the launch with zero velocity and height (h) obtained by trigonometry, while the maximum kinetic energy was produced when the ball reached its maximum velocity (v) and the height was zero; see Equation (1). Once the maximum speed was obtained, the centripetal force (F_c_) in Equation (2), which the trident withstood, was calculated, taking into account the mass (m) and length (r) of the telescopic system, as well as the aforementioned speed (v). The force exerted by the weight of the trident, the telescopic system and the fixed sphere (m) had to be added to the force that pulled the trident outwards. With these two forces, the result of the total force (F_T_) supported by the magnets of the trident could be obtained—see Equation (3)—which also depended on the position of the fingers of the trident at the moment of the launch.

(1) $$\frac{1}{2}{\mathrm{mv}}^{2}=\mathrm{mgh}$$

(2) $$F_{c}=m\frac{v^{2}}{r}$$

(3) $$F_{T}=\mathrm{mg}+m\frac{v^{2}}{r}$$

**Figure 6:**
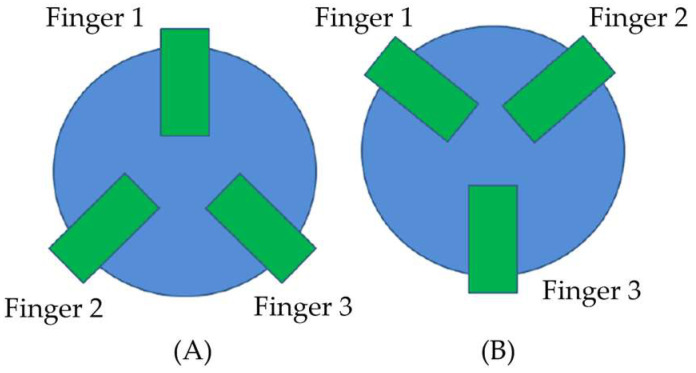
Launch positions: fingers’ geometric orientations in the dynamic test, (**A**) Two fingers back, (**B**) Two fingers front.

The force supported by each trident with the same contact point angles as in the previous static test was tested, also considering two different launch positions or geometric orientations of the fingers in the trident’s body (Figure 6). The first one had one of the fingers on the front and the other two fingers on the back with respect to the direction of the pendulum’s movement, and the second had the opposite fingers’ distribution, named (A) and (B), respectively, in the Figure 6.

**Figure 7:**
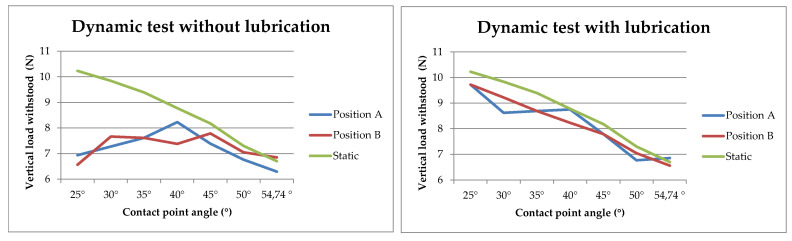
Tridents force dynamic test results (evaluation of contact point angle).

The dynamic test was carried out with and without lubrication between the magnets and the fixed sphere to analyze the influence of friction among the components during the testing. The final results obtained in the dynamic test can be seen in Figure 7.

Considering the force results obtained in the *static test*, a downward trend in the force supported by the trident magnets could be observed as the contact point angle increased. This was because the force supported had a vertical downward direction, so tridents with a smaller contact point angle had their fingers and, therefore, magnets oriented closer to vertical and would, therefore, withstand more force. When the tridents had a higher contact point angle, the direction of the force made it easier to separate them from the fixed sphere. After the static testing, it was, therefore, concluded that the trident with the smallest contact point angle performed best in this test.

In regard to the *dynamic testing* without lubrication, the results in Figure 7 showed that the tridents with the best behavior under these conditions were those in the range of 30° to 45° contact point angles. Tridents with a very small contact point angle would withstand a lower force since the three magnets were oriented in positions very close to the vertical, so that, in a dynamic pendulum test, the acceleration and velocity of the assembly would cause forces in directions almost perpendicular to those of the trident fingers that would force the magnets apart. On the other hand, when using tridents with a very high contact point angle, problems are also detected due to the vertical component of the forces as in the static test. Another problem generated was the so-called “leverage effect”. This effect, during the movement of the pendulum, and given that the fingers are very far apart, causes separation forces to be generated in the magnets at the back of the trident when sliding over the sphere, mainly due to the friction between the metal surface of the sphere and the magnets.

Under these test conditions, the tridents that behaved slightly better were, for launch position “A”, the trident with a 40° contact point angle design, while, for position “B”, the tridents that withstood the highest force were those with 30° and 45° angles.

In the case of the lubricated dynamic test, by eliminating much of the friction between the metal ball and the trident magnets, the performance of all tridents substantially improved, with all tridents withstanding higher forces. This positive influence on the system performance means that a thin film of lubricant can be formed in the dynamic test, worsening the system accuracy. However, in the target application, a volumetric verification of machine tools, the measurement was performed under static conditions, so that lubrication would not affect the accuracy of the system. The better performance of tridents with smaller contact point angles was again confirmed.

It could also be inferred from both tests, that the launch position had no influence, as apparently no trend could be identified that would allow a relationship to be established between each position and the contact point angle of the trident.

Therefore, based on the static and dynamic test results, under the hypothesis that the system works under intermediate lubrication conditions and looking for a compact design, it is, finally, concluded that the optimum contact point angle selected in the design of the trident would be 30 degrees.

### 3.4. Kinematic Coupling Design Versions

**Figure 8:**
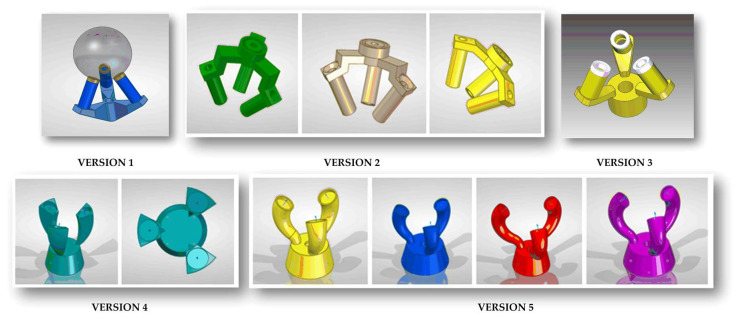
Trident’s design evolution.

The design of the kinematic coupling was modified during the development considering the functional and dimensional requirements, restrictions and design parameters explained in Section 3.2 and Section 3.3. Table 3 and Figure 8 show the different design versions (one to five), their main features and drawbacks identified in the design and in the testing of the prototypes.

A simulation was carried out to analyze how the deformation of the system and the variation of the design parameters could affect the possible displacements of the center of the fixed sphere due to the deformation of the trident. This would be critical and would modify the laser measurement of the sphere-to-sphere distance. To evaluate this point, the deformation in the trident was simulated by embedding the bottom of the trident body and applying a force perpendicular to the surface of each trident’s finger of 10 Newton, which exceeds the mass of the trident-arm assembly of 855 g.

The simulation results for different design iterations of version five are shown in Table 4. Design parameters changed in the simulation (grey shadowed in Table 4) and were the sphere–body distance to evaluate the effect of trident miniaturization, the trident’s body angle, the finger’s lower diameter and the finger’s arc radii.

The geometrical and dimensional design of the trident’s fingers was carried out trying to maximize the rigidity of the part and fulfilling the following functional requirements: (i) that the fingers do not invade the body area so that the minimum working angle can be reached without collisions, (ii) that the fingers of at least two tridents can interlace with each other.

The optimum finger shape is a circular extrusion along a central curve acting as an axis, which should start from the trident’s body in the Z-direction. This design did not trespass on the body area and allowed contact with the sphere perpendicular to its surface. This contact ensured that the magnet exerts an optimum attraction force and a steady and repeatable coupling is guaranteed. To achieve this in a simple way in the design of the fingers, a curve consisting of two arcs tangent to each other which can have different radii were used. It was observed in Table 4 that a smaller lower radius resulted in a better load resistance due to the curvature of the finger.

It was also observed that the trident design was stiffer the larger the diameter at the base of the finger, but it was important to leave enough space for the interlacing of the other fingers. In this regard, versions 5.1 and 5.2 behaved worse in deformation with values of 43.2 and 45.5 µm, and versions 5.3 and 5.4 drastically improved the behavior under load decreasing the deformation to 5.87 and 6.2 µm, respectively. Versions 5.5 and 5.6 presented lower deformations close to 2 µm, but without sufficient space between fingers.

In versions 5.7 and 5.8, the aim was to increase the intermediate space so that the fingers could interlace by increasing the angle of the body from 12 to 15 degrees and decreasing the diameter of the base of the finger (lower finger’s diameter) to 5.1 mm. Additionally, the finger length was decreased to the maximum for better strength, moving from a sphere–body distance of 36.9 to 29.1 mm. With both versions 5.7 and 5.8, minimum deformation values of 1.31 and 1.36 µm, respectively, were obtained. Version 5.7 was selected as the optimal final version of the trident design.

## 4. Prototyping and Experimental Results

Several prototypes of the trident designs were manufactured by 3D printing from all versions, varying the different dimensional parameters of our system during the validation phase. As mentioned in Section 3.4, the final optimal design was version 5.7, which minimized the trident’s deformation assuring performance.

**Figure 9:**
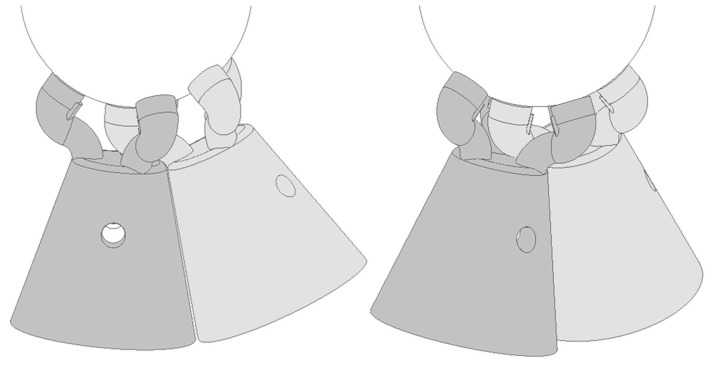
Detail of interlacing fingers in final design version.

The absence of interferences or collisions when the tridents interlaced inside each other were verifies with the prototypes, as in the gray-shaded tridents on the right side of Figure 9, and that the contact limit between the tridents’ bodies with the minimal angles needed in the application.

**Figure 10:**
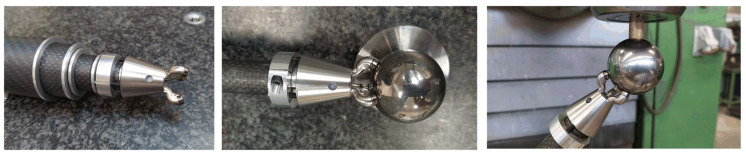
Final version of the multi-point kinematic coupling.

The final design of the kinematic coupling was mechanized (lathe machining and 5-axis milling) in stainless steel grade 304 L in a single part and was assembled in the telescopic system, see Figure 10, to complete the system validation in the laboratory and in the MTs of the application.

Finally, to characterize the behavior of the trident in the telescopic arm and to analyze how its positioning and registration on the fixed sphere affected the measurement repeatability of the telescopic arm, tests were carried out in the laboratory and in the workshop with the final version of the machined tridents assembled on the telescopic arms.

The laboratory test consisted of two kinematic supports located on the CMM bed at a distance of 470,822 mm between them. The end of the telescopic arm with the sphere, where the sensor head was located, was positioned on one kinematic support and the trident side of the arm was registered to a fixed sphere that was located on the second support. Once the distance was measured with the telescopic arm, the trident was removed from the fixed sphere and repositioned to measure the distance again. This procedure was repeated 10 times. Table 5 shows the results obtained for the mean, standard deviation and semi-range values of the sphere-to-sphere distance measured with the telescopic arms.

In the case of the *workshop test*, one side of the telescopic arm was mounted on a kinematic support located on the MT bed, and the trident’s side was registered to the fixed sphere mounted on the MT spindle nose (see Figure 1). The testing procedure was the same as in the laboratory test, measuring a distance between the spheres with the telescopic system, loosening and repositioning the trident for the next distance measurement. The procedure was repeated 10 times. The results obtained are shown in Table 5 with repeatability values below ±0.5 µm for the laboratory test, and ±1.2 µm for the workshop test.

## 5. Discussion

This paper provided the design proposal of a new multi-point kinematic coupling specially developed for a high precision multi-telescopic arm measurement system for the volumetric verification of machine tools with linear and/or rotary axes. Kinematic couplings design principles were applied in the development phase of the component, considering the requirements and restrictions of the interferometric telescopic measurement system, setting the main design parameters and generating several design alternatives. First, experimental results of the prototypes showed that they coped with the functionality required, avoiding collisions and enabling interlacing among the three kinematic couplings with minimal deformation and good repeatability values.

The main contributions of the work were the following:

- The trident has three fingers that generate a multi-point contact with the fixed sphere attached to the MT spindle nose. This was achieved with three magnets located at the ends of the fingers. They have enough force not to be detached from the fixed sphere along the MT travel in the different axis at high feed rates (up to 20 m/min). It also generates repulsion forces among the other trident’s fingers to avoid collisions or interferences that would hinder the correct working of the telescopic arms set.
- The design of the fingers assures their interlacing and the body allows the fingers not to collide up to a certain angle depending on the range of the MT to be verified. The fingers are short to minimize the distance between the fixed sphere attached to the MT spindle’s nose and the retroreflector. This allows maximizing the accuracy of the distance measurement.
- The design process took into account experimental studies to define the optimal contact point angle of the fingers, concluding that under the hypothesis that the system works in intermediate lubrication conditions and looking for a compact design, an angle of 30 degrees was chosen.
- The trident design was modified during the development considering the functional and dimensional requirements throughout different design versions, analyzing its main features and drawbacks.
- The final design also minimizes the deformation of the trident that could lead to a relative movement between the center of the sphere and the trident, as this would include an error in the laser measurement of the sphere-to-sphere distance.
- The final design of the kinematic coupling was mechanized by lathe machining and five-axis milling in stainless steel grade 304 L in a single part. Finally, repeatability tests were performed in the laboratory and in the workshop of the trident assembled on the telescopic arm. The results obtained showed repeatability values lower than 0.5 µm for the laboratory test, and 1.2 µm for the workshop test.

## 6. Conclusions

An accurate and robust multi-point kinematic coupling was designed, manufactured and tested to allow the simultaneous verification of machine tools with three telescopic arms that are registered at the same time to a sphere fixed on the MT spindle nose. It could be concluded that the selected trident’s geometry allows the fingers to interlace with each other so that three systems can work simultaneously up to a very small trident body angle, making it possible to address the simultaneous verification of most existing MTs.

The integration of three or more high precision telescopic measurement systems in the verification of MTs would allow to improve the accuracy of the verification and to reduce the working time, so that thermal drifts would have significantly less influence. This achievement was possible due to the inclusion in the measurement system of the multi-point kinematic coupling designed in this work. Therefore, a substantial contribution to the development of MT volumetric verification techniques could be derived from the work presented.

## Figures and Tables

**Table 1:** Multi-point kinematic coupling design parameters.

Device	Item	ID	Design Parameter	Unit
Group 1	Trident’s body	1	Trident’s body angle	degree
		2	Trident’s body height	mm
	Trident’s finger	3	Finger’s upper arc radii	mm
		4	Finger’s lower arc radii	mm
		5	Finger’s upper diameter	mm
		6	Finger’s lower diameter	mm
Group 2	Fixed sphere	7	Fixed sphere diameter	mm
	Magnet	5	Magnet diameter	mm
		8	Magnet height	mm
	Trident–Sphere	9	Contact point angle	degree
		10	Fixed sphere–trident body distance	mm

**Table 2:** Tridents force static tests results (evaluation of contact point angle).

**Contact Point Angle (Degrees)**	25	30	35	40	45	50	54.74
**Force (kg)**	1.04	1.00	0.96	0.90	0.83	0.74	0.68

**Table 3:** Trident’s design versions, features and drawbacks.

Prototype Version	Features/Advantages	Drawbacks
1	Trident divided in two parts, bolted body and fingers Simple finger and body geometry easy to machine	Interferences detected in the elements Collisions among fingers and tridents’ bodies
2	Trident divided in two parts, bolted body and fingers Simple finger and body geometry easy to machine Three different trident heights for easy interlacing Body with rectangular sections easy to machine	Collisions due to rectangular sections Rotation is avoided No tridents interlacing as they are at different angles
3	Trident divided in two parts, bolted body and fingers Smooth and curved design lines in the trident body Design of the fingers support above the body trying to avoid collision between the fingers and the bodies	Interference between elements Collisions among fingers and not between tridents’ bodies Lightweight finger support design
		Difficult machining Lack of rigidity of the assembly
4	Trident in one single part (body and fingers) Smoother, curved design lines in the trident body	Collisions due to straight lateral finger surfaces Lightweight finger-to-body design
	Fingers come out vertically from the body to reduce collisions	Lack of rigidity of the assembly Medium difficulty in machining
	Simple design for easy turning and milling	
5	Same trident concept as version 4 The fingers came out completely vertical from the body thanks to an arch at the bottom Reduction in the finger lower radius versus its upper radius Rounded finger’s lateral surface Trident fingers interlace easily in pairs	Limited miniaturization to enable the three trident’s fingers interlacing simultaneously Trident fingers interlace in pairs, but triple interlacing is hardly needed in operation Complex lathe machining and 5-axis milling for trident manufacturing are required

**Table 4:** Trident design parameters and deformation simulation.

	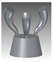	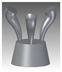	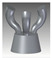	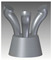	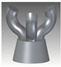	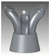	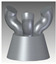	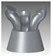
**Version**	5.1	5.2	5.3	5.4	5.5	5.6	5.7	5.8
Fixed sphere diameter (mm)	38.1	38.1	38.1	38.1	38.1	38.1	38.1	38.1
Magnet diameter (mm)	6	6.5	6.5	6.5	6.5	6.5	6.5	6.5
Magnet height (mm)	3	3	3	3	3	3	3	3
Contact point angle (°)	30	30	30	30	30	30	30	30
Sphere–body distance (mm)	36.9	36.9	36.9	36.9	36.9	36.9	29.1	29.1
Finger arc radii (mm)	9.4/5	5.6/15	8.6/5	4.5/15	8.7/5	3.5/15	5.7/4	3.5/8.5
Trident body angle (°)	12	12	12	12	12	12	15	15
Finger upper diameter (mm)	6.5	6.5	6.5	6.5	6.5	6.5	6.5	6.5
Finger lower diameter (mm)	2	2	4	4	6	6	5.1	5.1
Trident body height (mm)	12	12	12	12	12	12	12	12
Deformation (µm)	43.2	45.5	5.87	6.2	1.92	2.07	1.31	1.36

**Table 5:** Telescopic system measurement repeatability considering trident re-positioning.

Telescopic Arm	Laboratory			Workshop		
	Mean (mm)	Std (mm)	Semi-Range (mm)	Mean (mm)	Std (mm)	Semi-Range (mm)
1	470.8224	0.0003	0.00045	572.3533	0.0008	0.0012
